# Frontal Polymerization-Enabled Rapid Fabrication of Gelatin-Containing Hydrogels with Good Mechanical and Biological Properties

**DOI:** 10.3390/gels12060547

**Published:** 2026-06-19

**Authors:** Fucheng Li, Weixiong Yuan, Yonglin Chen, Chang Liu, Cai-Feng Wang, Su Chen

**Affiliations:** State Key Laboratory of Materials-Oriented Chemical Engineering, College of Chemical Engineering, Nanjing Tech University, Nanjing 210009, China; imlfc@foxmail.com (F.L.); yuanweixiong22@163.com (W.Y.); chenyonglin1998@163.com (Y.C.); chensu@njtech.edu.cn (S.C.)

**Keywords:** gelatin hydrogels, frontal polymerization, mechanical properties, biocompatibility, antibacterial activity, carbon dots

## Abstract

A time-saving approach to gelatin-based hydrogels with versatile properties is highly desirable. Herein, we report the rapid fabrication of new gelatin-containing hydrogels with favorable mechanical properties, biocompatibility and antibacterial capability. Frontal polymerization (FP) of acrylic acid (AA), acrylamide (AM), hydroxypropyl acrylate (HPA) with gelatin methacryloyl (GelMA) enables the rapid formation of multifunctional hydrogels within 7 min, providing a highly efficient route for gelatin-based hydrogel fabrication. The effect of GelMA content on FP features and hydrogel properties was systematically investigated. The resultant hydrogels show attractive collective properties with tensile strength up to 101.3 kPa, elongation at break up to 227.7%, cell viability of 96% after 24 h, and antibacterial activity against *S. aureus* (92.2%). In addition, the FP of the hydrogels with use of forsythia-derived carbon dots (F-CDs) as bioactive nanofillers is explored, conferring the hydrogels with enhanced mechanical performance and biocompatibility, demonstrating the applicability of this FP strategy upon incorporating functional additives. This work provides a simple and effective approach for the rapid preparation of gelatin-containing hydrogels with versatile functions promising for biomedical applications such as wound healing and tissue engineering.

## 1. Introduction

Hydrogels are a class of soft materials possessing three-dimensional network structures formed through chemical or physical crosslinking [1,2,3,4]. These soft materials have found broad applications in biomedical, industrial, agricultural and environmental fields [5,6,7,8,9]. Among them, bio-based hydrogels derived from natural polymers have attracted particular attention owing to their biocompatibility, low toxicity and inherent bioactivity [10,11,12]. Gelatin, a denatured product of collagen, is one such widely used bio-based material. It exhibits excellent biocompatibility and biodegradability [13], as well as an extracellular matrix-like structure, making it suitable for tissue engineering, wound healing and regenerative medicine [14,15,16]. Its bioactive functional sites further support cell adhesion and tissue remodeling [17]. However, physically crosslinked gelatin hydrogels suffer from several limitations that restrict their broader application. They are thermally unstable at physiological temperatures (25–37 °C) [13,18,19], mechanically weak and prone to irreversible deformation under repeated stress [20]. In addition, gelatin lacks intrinsic antimicrobial activity and its high-water content can result in uncontrolled swelling or rapid degradation, compromising performance in biomedical contexts [14,18,21].

To overcome these limitations, the incorporation of synthetic polymers with gelatin via physical blending or chemical crosslinking has been developed [20,22]. For instance, gelatin can be chemically modified with methacrylate groups to produce gelatin methacryloyl (GelMA) [23,24]. GelMA has double bonds that can polymerize with other monomers, enabling the availability of denser and mechanically tunable hydrogels with collective or synergistic properties better meeting the requirements for practical applications [17,18,21,25,26]. However, conventional GelMA hydrogels still face limitations such as slow polymerization, uneven network formation and restricted performance [27,28]. Rapid and controlled polymerization strategies for the fabrication of GelMA hydrogels with versatile properties are highly desirable.

Frontal polymerization (FP) is an efficient polymerization method that relies on a self-sustaining reaction front [29,30]. Once initiated, the reaction zone propagates through the monomer mixture and rapidly converting monomers to polymers, driven by the enthalpy of polymerization itself, without need of an external energy source. Compared with conventional polymerization methods, FP offers significant advantages, including ultrafast synthesis rates, low energy consumption, simple operation, and uniform product formation. It also enables high monomer conversion and better control over microstructure, which are particularly beneficial for preparing high-performance hydrogels [30]. This technique has been successfully applied to fabricate a wide range of functional materials, including hydrogels, resins, thermosetting polymers, foams, nanocomposites and gradient or patterned polymers [31,32]. Two recent studies have also demonstrated its potential for gelatin-based hydrogels. Specifically, FP was employed to produce GelMA-containing hydrogels with enhanced mechanical strength and self-healing via interpenetrating polymer networks [20], whilst we fabricated hydrogel microfluidic chips with improved mechanical stability and biocompatibility via FP [33].

In this work, we report rapid fabrication of new gelatin-containing hydrogels featuring excellent mechanical properties, biocompatibility and antibacterial capability via FP. A monomer system composed of acrylic acid (AA), acrylamide (AM), hydroxypropyl acrylate (HPA) and gelatin methacryloyl (GelMA) was designed to implement FP, to rapidly generate the hydrogels (denoted as PAAH-GelMA) within 7 min. The influence of GelMA ratio on FP features during the synthetic process and on the properties of the resulting hydrogels including morphology, mechanical performance, and water swelling were systematically investigated. The biocompatibility and antibacterial activity of the hydrogels were also indicated. Furthermore, we explored the FP of gelatin-containing hydrogels with use of low-toxicity forsythia-derived carbon dots (F-CDs) as nanofillers, to yield CDs/PAAH-GelMA hydrogels with enhanced mechanical strength and biocompatibility. The results demonstrate the synergistic enhancement of multi-component copolymerization and nanofiller reinforcement via the time- and energy-saving approach of FP, promising the rapid preparation of gelatin-containing hydrogels with versatile functions.

## 2. Results and Discussion

### 2.1. Preparation of PAAH-GelMA Hydrogels via Frontal Polymerization

In this work, GelMA was synthesized via methacrylation of gelatin and used as a monomer together with AM, AA and HPA to carry out FP for rapid generation of gelatin-containing hydrogels with versatile functions. Firstly, gelatin was methacrylated to synthesize GelMA, providing a chemically crosslinkable backbone with excellent biocompatibility (Figure S1a,b). The grafting of methacrylate groups and the structural integrity of GelMA were verified by proton nuclear magnetic resonance (^1^H NMR) and Fourier transform infrared (FTIR) spectroscopy (Figures S1c and S2). Specifically, the ^1^H NMR spectrum showed new peaks at δ = 5.44 and 5.67 ppm (vinyl protons, C=CH_2_) and δ = 1.92 ppm (methyl protons), along with a significant intensity decrease in the lysine methylene proton peak at δ = 3.01 ppm. Based on the NMR integral changes, the degree of methacrylation (*DM*) was calculated to be approximately 63%, indicating a high density of reactive sites for subsequent network formation. Additionally, the FTIR spectrum displayed characteristic peaks at 3321, 1634, and 1540 cm^−1^, confirming the preservation of the peptide backbone.

**Figure 1:**
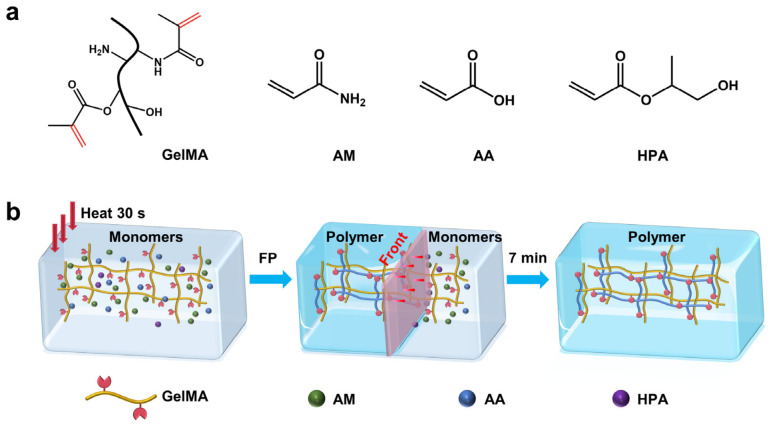
(**a**) Chemical structure of monomers designed for the reaction system and (**b**) schematic diagram of the synthetic procedure for the preparation of PAAH-GelMA hydrogels via FP. The schematic diagram shows interpenetrating polymeric network of the product, where gelatin (brown lines) and FP-derived polymer (blue lines) are covalently linked via reactive methacryloyl groups (red lines/shapes) of GelMA.

Conventional GelMA hydrogels often suffer from slow polymerization, uneven network formation in bulk samples, limited mechanical performance and lack of multifunctions [19,20,28]. To overcome these limitations, a multi-monomer system consisting of GelMA, AM, AA and HPA was designed to conduct horizontal FP, to rapidly fabricate PAAH-GelMA hydrogels (Figure 1a). In this system, AM and AA function as highly reactive comonomers, releasing substantial heat during polymerization to sustain self-propagation of the FP front and promote uniform network formation throughout the precursor solution. Meanwhile, HPA was incorporated as a thermodynamic regulator to moderate the reaction rate and heat release during FP [34]. The hydroxyl groups on its side chains induce chain-transfer effects, thereby suppressing bubble formation and preventing thermal denaturation of the GelMA backbone. Controlled macromolecular GelMA content and the use of ethylene glycol (EG) as solvent also helped to reduce thermal gradients and balance thermal expansion with chemical shrinkage. To further stabilize the network structure, poly(ethylene glycol) diacrylate (PEGDA 1000) was introduced as the chemical crosslinker. These designs minimized residual stresses, micro-cracks, and structural defects arising from exothermic reactions. *N*,*N*,*N*′,*N*′-tetramethylethylenediamine (TMEDA) and ammonium persulfate (APS) were employed for redox initiation. As illustrated in Figure 1b, upon localized thermal triggering within 30 s, a self-sustaining polymerization front was generated and propagated steadily through the precursor solution. Once the polymerization front was formed, no external heat source was required thereafter, while the front continued to propagate autonomously to achieve the complete conversion of monomers into polymers, driven by the exothermic heat by the reaction system itself. Using this FP strategy, PAAH-GelMA hydrogels could be rapidly fabricated within 7 min.

**Figure 2:**
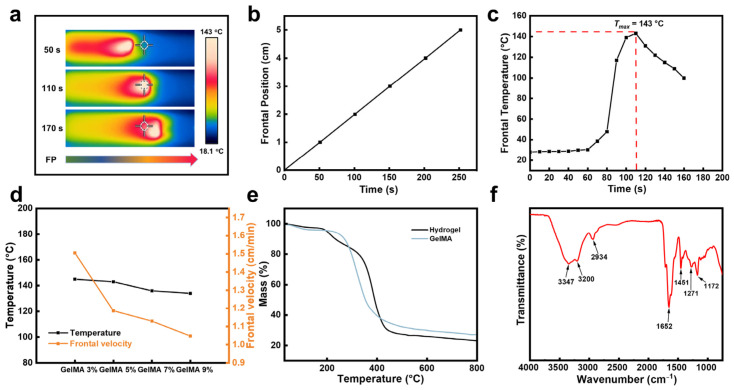
(**a**) Infrared thermal images captured at 60 s intervals for the propagation during the FP process. (**b**) The plot of frontal position versus time during the FP process. (**c**) Temperature–time curve during the FP process. (**d**) Frontal velocity and *T*_max_ as a function of GelMA mass fraction. (**e**) TGA curves of GelMA and PAAH-GelMA hydrogels. (**f**) FTIR spectrum of the PAAH-GelMA hydrogels. Unless otherwise specified, the mass fraction of GelMA in the hydrogels is 5%.

The occurrence of pure FP without spontaneous polymerization could be confirmed by two main features, i.e., frontal velocity and thermal profiles [32]. First, infrared thermal imaging revealed a mild and steady propagation process, during which the reaction front continuously advanced through the precursor solution (Figure 2a). The frontal position exhibited a linear dependence on time, indicating a constant propagation velocity (Figure 2b). Second, the temperature at a fixed position in the reaction system displayed a typical FP thermal profile during propagation (Figure 2c). Prior to the arrival of the reaction front, the temperature remained nearly constant. As the front passed the monitored position, the temperature rapidly increased to a maximum value (*T*_max_) and subsequently decreased gradually. This characteristic rise-and-fall behavior further confirms the stable and self-sustaining nature of the FP process.

We further investigated the effect of GelMA content on FP kinetics (Figure 2d and Figure S3). All frontal position–time curves exhibited good linearity, confirming the occurrence of pure FP in all reaction systems. As the mass fraction of GelMA increased from 3% to 9%, the front velocity decreased from 1.50 to 1.05 cm/min and *T*_max_ decreased from 145 to 134 °C. The results indicate that the increase in the GelMA content causes a decrease in the front velocity and temperature in the FP process. This trend is likely due to the large molecular size of GelMA, which requires more heat to initiate polymerization. Higher GelMA content consumes more thermal energy, thereby slowing the reaction and reducing the maximum temperature.

Then we investigated the composition of the product produced by FP. Thermal stability of the hydrogels was assessed by thermogravimetric analysis (TGA) (Figure 2e). The pure GelMA exhibited an initial weight loss of 4.5% in the range of 30–190 °C, arising from the loss of structured water hydrogen-bonded to the protein molecules [35], followed by a major thermal decomposition stage within 214–600 °C for the gelatin macromolecule. In comparison, the PAAH-GelMA hydrogel commenced decomposition at 190 °C and displayed a second degradation stage at 287 °C. The distinct two-stage decomposition confirms the formation of interpenetrating polymeric network hydrogel system as illustrated in the right of Figure 1b. The FTIR spectrum of the PAAH-GelMA hydrogel shows characteristic absorption peaks at 3347 and 3200 cm^−1^ (N–H and O–H stretching), 2934 cm^−1^ (C–H stretching), 1652 cm^−1^ (C=O stretching), 1271 cm^−1^ (N–H bending) and 1172 cm^−1^ (C–O–C stretching in the ester group from HPA) (Figure 2f). These results indicate that FP offers a straightforward and efficient approach to fabricate hydrogels containing GelMA.

### 2.2. Microscopic Morphology, Mechanical Properties and Swelling Behavior of PAAH-GelMA Hydrogels

**Figure 3:**
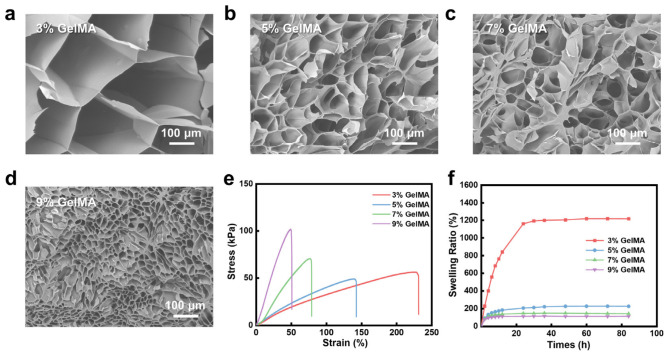
(**a**–**d**) SEM images of the hydrogels with varying GelMA contents. (**e**) Stress–strain curves of the hydrogels with different GelMA mass fractions. (**f**) Swelling ratio in water versus time across different GelMA mass fractions.

The microstructure of the PAAH-GelMA hydrogels was examined using scanning electron microscopy (SEM). As shown in Figure 3a–d, the hydrogels exhibit an interconnected microporous structure with a relatively uniform pore distribution. Notably, as the GelMA mass content increases from 3% to 9%, the average pore diameter of the hydrogels decreases significantly from 330 to 32 μm (330, 84, 71 and 32 μm for the samples prepared with 3%, 5%, 7% and 9% GelMA, respectively). This reduction is mainly attributed to the introduction of GelMA macromolecules, which effectively increases the crosslinking density and leads to a more compact network architecture. GelMA also participates in the FP process through its methacryloyl functionalities, and variations in its content influence polymer network formation and consequently affect the resulting microstructure and physicochemical properties of the hydrogels.

The mechanical properties of the hydrogels with varying GelMA content were also evaluated. Figure 3e and Figure S4 display the tensile stress–strain curves and the corresponding Young’s moduli. With increasing GelMA content, the tensile strength improves from 56.1 to 101.3 kPa and the Young’s modulus surges from 23.8 to 233.2 kPa, while the elongation at break decreases from 227.7% to 49.3%. These mechanical results are consistent with the SEM analysis. The increased crosslinking density induced by GelMA incorporation leads to a more compact network structure, which facilitates more efficient stress transfer throughout the hydrogel matrix and restricts polymer-chain mobility. As a result, the hydrogels exhibit enhanced tensile strength and stiffness. However, the reduced chain mobility and decreased pore size of microporous structure limit the ability of the polymer chains to undergo large-scale deformation under tensile loading, leading to a reduction in elongation at break.

Furthermore, the swelling kinetics of the FP-synthesized PAAH-GelMA hydrogels were evaluated in deionized water at room temperature using gravimetric analysis (Figure 3f). Hydrogels with different GelMA mass ratios exhibited an initial increase in swelling, followed by a gradual approach to equilibrium. As expected, the equilibrium swelling ratio demonstrated a continuous downward trend from 1216% to 113% as the GelMA content increased. This reduction is fundamentally ascribed to the introduction of GelMA, which significantly enhances the crosslinking density of the polymeric network. Consequently, this denser network architecture restricts pore expansion, thereby diminishing the overall water retention capacity of the hydrogels. Based on the present results, key design considerations for multi-component FP systems include maintaining sufficient reaction exothermicity to sustain stable front propagation, minimizing bubble formation to preserve structural uniformity, and tuning network architecture through appropriate control of crosslinking density to achieve the desired porosity and microstructure. Such tunable microporous morphology with favorable swelling behavior and mechanical properties, makes the hydrogels promising candidates for multifunctional soft materials in applications such as cell culture, tissue engineering or wound dressing.

### 2.3. Biocompatibility and Antibacterial Properties of PAAH-GelMA Hydrogels

**Figure 4:**
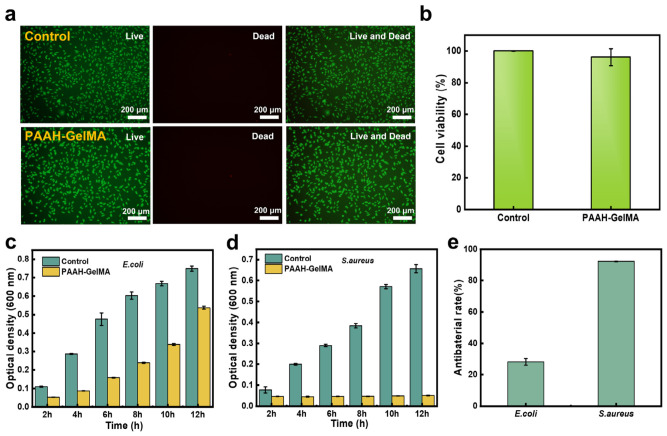
(**a**) Fluorescence images of Live/Dead staining for L929 fibroblasts cultured in the control medium and PAAH-GelMA hydrogels extract for 24 h. (**b**) Cytocompatibility evaluation of hydrogels via CCK-8 assay. Time-dependent optical density (OD600) of (**c**) *E. coli* and (**d**) *S. aureus* suspensions co-cultured with the samples. (**e**) Antibacterial rates of the PAAH-GelMA hydrogels against *E. coli* and *S. aureus*.

Given prospective biomedical uses including cell culture and tissue engineering, it is imperative that the soft materials possess reliable biosafety and the ability to prevent bacterial infection. Therefore, their biocompatibility and antibacterial activity were systematically evaluated. To evaluate the biocompatibility of the PAAH-GelMA hydrogels, L929 fibroblast cells were cultured with the hydrogels extract for 24 h, followed by Live/Dead staining to assess cell viability and toxicity. As shown in Figure 4a, Live/Dead staining revealed abundant viable cells with normal morphology (green fluorescence) and negligible dead cells (red fluorescence). To further quantify cell proliferation and gain deeper insight into collective cellular behavior, the Cell Counting Kit-8 (CCK-8) assay was performed. As shown in Figure 4b, the hydrogels exhibit a high cell viability of 96%, demonstrating their excellent biocompatibility and negligible cytotoxicity.

The antibacterial performance of PAAH-GelMA hydrogels was tested against *E. coli* and *S. aureus* over 12 h. Compared to the phosphate-buffered saline (PBS) control, the hydrogels significantly reduced OD600 values over time (Figure 4c,d), achieving inhibition rates of 28.1% for *E. coli* and 92.2% for *S. aureus* (Figure 4e). The coated plate method was further utilized to measure the antibacterial efficacy of the hydrogels (Figure S5). The resulting images confirmed a distinct reduction in bacterial colony formation, particularly for *S. aureus*. Mechanistically, the antibacterial activity is probably attributed to a synergistic mechanism that AA-derived carboxyl groups create an acidic microenvironment to disrupt cell membranes [36], while AM-derived groups protonate into cationic -NH_3_^+^ sites that electrostatically rupture negatively charged bacteria [37]. In addition, the hydrated polymer network may hinder bacterial adhesion and nutrient transport, thereby enhancing antibacterial efficacy [38]. Notably, the hydrogels exerted stronger antibacterial effects on *S. aureus* than on *E. coli*. This difference arises from structural variations between Gram-positive and Gram-negative bacteria. Without an outer membrane, Gram-positive bacteria are more vulnerable to external environmental stresses [39]. Overall, the PAAH-GelMA hydrogels demonstrate effective antibacterial activity, excellent biocompatibility and low cytotoxicity, highlighting their potential for biomedical applications, particularly as wound dressings.

### 2.4. Frontal Polymerization of CDs/PAAH-GelMA Hydrogels

**Figure 5:**
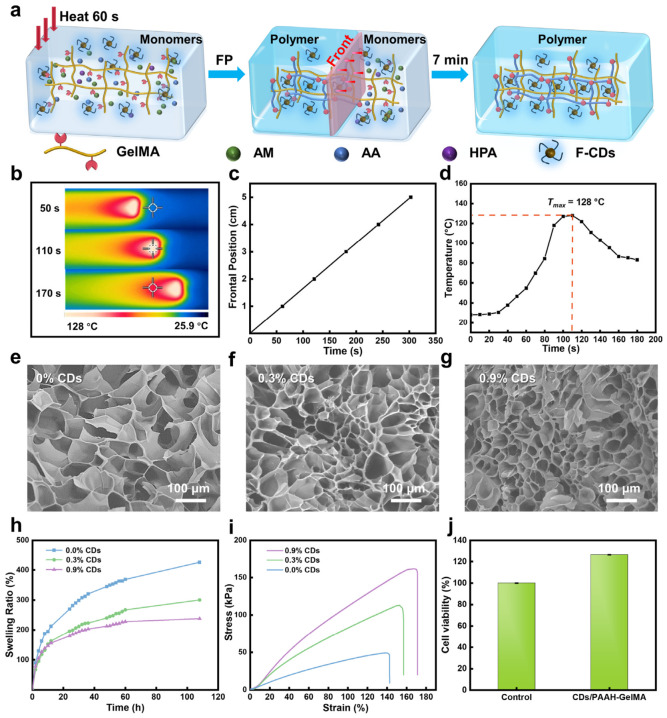
(**a**) Schematic illustration of the FP process for preparing CDs/PAAH-GelMA hydrogels. (**b**) Infrared thermal images during FP propagation. (**c**) Front position versus time curve. (**d**) Temperature–time profile. (**e**–**g**) SEM images of hydrogels with 0, 0.3 and 0.9% mass content of F-CDs. (**h**) Swelling ratios versus time for hydrogels with different F-CDs mass fractions. (**i**) Stress–strain curves for hydrogels with different F-CDs mass fractions. (**j**) Relative viability of L929 fibroblasts cultured in the CDs/PAAH-GelMA hydrogels extracts for 24 h, assessed via CCK-8 assay. Unless otherwise specified, the mass fractions of F-CDs and GelMA in the hydrogels are 0.9% and 5%, respectively.

To evaluate the applicability of the established FP process upon the introduction of external active agents, forsythia-derived carbon dots (F-CDs) were selected as functional additives. Forsythia is well known as a herbal medicine useful for treating issues such as inflammation, fever, and ulcers, and the derived F-CDs possess bioactive activity and nanofiller reinforcement capability [40,41]. They were subsequently incorporated into the precursor to fabricate CDs/PAAH-GelMA hybrid hydrogels via FP (Figure 5a). The presence of F-CDs did not disrupt the fundamental FP behavior. As shown in Figure 5b, infrared thermal imaging captured a uniform and stable thermal front advancing continuously from left to right. The front position versus time plot remained highly linear, indicating a constant propagation velocity of 0.99 cm/min (Figure 5c). Furthermore, the corresponding temperature–time profile exhibited a clear maximum temperature of 128 °C (Figure 5d). Collectively, these observations confirm that the incorporation of F-CDs preserves a well-defined, self-sustaining FP process. We noted that the frontal propagation failed when the F-CDs content was increased to 1.2% in the current monomer formulation (Figure S6).

The incorporation of F-CDs led to a more compact network architecture. SEM images (Figure 5e–g) show that the average pore size of the hydrogels drops from 84 to 33 μm with the F-CDs content increased (specifically, 84, 68 and 33 μm for 0%, 0.3% and 0.9% F-CDs, respectively). Consistently, the equilibrium swelling ratio decreased from 426% to 238% (Figure 5h). These structural changes are primarily attributed to the abundant hydrophilic functional groups (e.g., carboxyl and hydroxyl groups) on the surface of the F-CDs. These groups act as physical crosslinking centers by forming dynamic hydrogen bonds with the PAAH-GelMA polymeric chains, thereby increasing the overall crosslinking density and yielding a more compact network.

Benefiting from this crosslinked network, the mechanical strength of the hydrogels was greatly improved. As reflected in the stress–strain curves and corresponding Young’s modulus bar charts (Figure 5i and Figure S7), increasing the F-CDs content markedly improves the tensile strength from 49.1 to 161.5 kPa, reaching approximately three times that of the pristine hydrogel, while the elongation at break increased from 142.4% to 171.2%, corresponding to approximately 1.2 times its original value. The simultaneous improvement in stiffness and extensibility indicates that F-CDs effectively serve as nano-reinforcement fillers, possibly promoting molecular entanglement and facilitating efficient energy dissipation under mechanical stress. These findings demonstrate that F-CDs serve as multifunctional additives in the FP-fabricated hydrogels by regulating the network architecture and reinforcing the mechanical properties of the resulting hydrogels.

Beyond mechanical reinforcement, F-CDs also improved the biological performance of the hydrogels. A CCK-8 assay with L929 fibroblasts cultured in hydrogels extracts for 24 h shows a cell proliferation rate of 126% relative to the PBS control (Figure 5j). This result not only demonstrates the excellent biocompatibility of the composite hydrogels but also indicates that the incorporation of F-CDs actively promotes cell proliferation compared to the pristine hydrogels without CDs. F-CDs with abundant surface functional groups derived from the herbal forsythia have excellent biocompatibility and bioactivity [41], which can improve the cellular microenvironment and facilitate cell proliferation [42]. Overall, these findings highlight that PAAH-GelMA hydrogels, rapidly fabricated via FP, provide a versatile and robust platform for incorporating functional nanomaterials such as F-CDs. The intrinsic network structure of the PAAH-GelMA matrix, combined with the synergistic reinforcement from F-CDs, results in enhanced mechanical performance and bioactivity, substantially broadening the potential of these composite hydrogels for demanding biomedical applications. While these materials exhibit favorable mechanical and biological characteristics, their in vitro and in vivo degradation behaviors as well as overall lifecycle remain to be comprehensively investigated in future research.

## 3. Conclusions

In conclusion, we developed a new gelatin-containing hydrogel system suitable for frontal polymerization using a monomer mixture of AA, AM, HPA, and GelMA. The polymerization process was self-sustaining and highly energy-efficient, enabling hydrogel formation within only 7 min, thereby effectively addressing the inherent slow gelation drawback typically associated with conventional GelMA hydrogels. We systematically investigated the effect of GelMA content on FP features and hydrogel properties. An increase in the GelMA content leads to a decrease in frontal velocity and temperature, along with a more compact network structure with smaller pore sizes, higher crosslinking density, improved mechanical strength, and reduced elongation at break. As such, we obtained gelatin-containing hydrogels possessing tunable microstructures, adjustable swelling behavior, and satisfactory mechanical properties (tensile strength up to 101.3 kPa and elongation at break up to 227.7%), biocompatibility (L929 fibroblast viability of 96%), and antibacterial activity against *S. aureus* (92.2%), solving the limitations of gelatin hydrogels like insufficient mechanical strength and limited functions. Furthermore, the established FP process is also applicable upon the introduction of external active agents. The frontal polymerization upon the use of forsythia-derived carbon dots as bioactive nanofillers yielded hybrid hydrogels. The hybrid hydrogels have 3 and 1.2 times higher tensile strength and elongation than the pristine hydrogels, and excellent cytocompatibility towards L929 fibroblast cells with cell viability of 126%. Continuous effort is needed for further optimizing the FP process to facilitate larger-scale production of these multifunctional gelatin-based hydrogels. Overall, this work paves a way towards rapid fabrication of multifunctional gelatin-containing hydrogels potentially useful for various biomedical applications.

## 4. Materials and Methods

### 4.1. Materials

Acrylic acid (AA, ≥99%), acrylamide (AM, ≥99%), hydroxypropyl acrylate (HPA, ≥98%), ethylene glycol (EG, ≥99%), poly(ethylene glycol) diacrylate (PEGDA 1000), methacrylic anhydride (MA, ≥94%), *N*,*N*,*N*′,*N*′-tetramethylethylenediamine (TMEDA, ≥99%), ammonium persulfate (APS, ≥98%) and gelatin (type A, from porcine skin) were purchased from Shanghai Aladdin Biochemical Technology Co., Ltd. (Shanghai, China) or Shanghai Macklin Biochemical Co., Ltd. (Shanghai, China). All chemicals were used as received without any further purification. L929 cells were obtained from Procell Life Science Co., Ltd. (Wuhan, China). Forsythia-derived carbon dots (F-CDs) were prepared by a magnetic heating method according to our previous work [41].

### 4.2. Preparation of Gelatin Methacrylate (GelMA)

GelMA was synthesized based on a reported method [17] with minor modifications. In a typical procedure, gelatin powder (10 g) was completely solubilized in 100 mL of Dulbecco’s Phosphate-Buffered Saline (DPBS), with the thermal environment maintained at 50 °C. After complete dissolution, methacrylic anhydride (8 mL) was steadily dispensed into the reactor using a peristaltic pump. The methacrylation process was then sustained at 50 °C for a duration of 3 h. To eliminate unreacted residues, the crude mixture was enclosed within semi-permeable membranes (8–14 kDa) and subjected to a seven-day dialysis process against deionized water. This purification was conducted under light-deprived conditions, involving daily solvent exchanges. The dialyzed product was then filtered to remove residual impurities, lyophilized, and stored at −20 °C until further use. ^1^H NMR spectroscopy was utilized to assess the methacrylation extent of the resulting GelMA. The exact degree of methacrylation (*DM*) was subsequently computed via the following expression:

(1) $$DM\;=\;(1-\frac{A_{\mathrm{GelMA}}}{A_{\mathrm{gelatin}}})\times 100\%$$

where *A*_GelMA_ and *A*_gelatin_ represent the integrated areas of the lysine methylene proton peaks (typically around 2.9 ppm) in the 1H NMR spectra of GelMA and unmodified gelatin, respectively. Prior to the calculation, these peak areas were normalized against the inert aromatic proton signals of phenylalanine (typically around 7.3 ppm) as an internal reference.

### 4.3. Frontal Polymerization of PAAH-GelMA Hydrogels 

The PAAH-GelMA hydrogels were synthesized via FP using EG as the sole solvent without the addition of water. First, AM, AA and HPA were dissolved in EG. Then GelMA was added at varying mass fractions (0 to 9%) and magnetically stirred at 30 °C until a clear and homogeneous mixture was obtained. After the solution cooled to room temperature, APS and PEGDA were added and stirred until fully dissolved. Immediately prior to initiation, TMEDA was added dropwise while stirring quickly for about 15 s. The APS:TMEDA molar ratio was 4:1. The precursor solution was poured into a quartz boat. To initiate the FP process, localized heating was applied to one end of the mold using a soldering iron (100 °C) for approximately 30 s until a distinct polymerization front emerged. After removing the heat source, the polymerization front propagated spontaneously through the unpolymerized region, completing the formation of the hydrogel in about 7 min. The typical precursor mass composition was set as follows: AM = 24.2%, AA = 15.2%, HPA = 5.6%, GelMA = 5%, EG = 49.3%, PEGDA = 0.4% and APS = 0.3%. After polymerization was complete, the hydrogel surface was gently rinsed with EG to remove any residual precursors. The samples were subsequently demolded and immersed in deionized water for 7 days (with daily water replacement) to extract unreacted monomers and soluble fractions. Finally, the hydrogels were freeze-dried to get aerogel-like samples for further characterization.

### 4.4. Preparation of CDs/PAAH-GelMA Hydrogels via Frontal Polymerization

The CDs/PAAH-GelMA hydrogels were prepared using a procedure similar to that of the PAAH-GelMA hydrogels, with the addition of F-CDs. Briefly, F-CDs were first dispersed in EG via ultrasonication. Subsequently, AM, AA and HPA were added and stirred until fully dissolved. GelMA was incorporated at the desired mass fraction, followed by APS and PEGDA. TMEDA was added last to initiate the redox system. The precursor solution was then transferred into a quartz boat or cast into a mold. To initiate the FP process, localized heating was applied to one end of the mold using a soldering iron (110 °C) for approximately 1 min until a distinct polymerization front emerged. Upon removal of the heat source, the front propagated spontaneously through the remaining precursor, completing the polymerization in approximately 7 min. Following polymerization, the hydrogels surface was rinsed with EG to remove residual precursors. The samples were gently removed from the molds and soaked in deionized water for 7 days, with the water replaced daily to remove any unreacted monomers. Finally, the hydrogels were lyophilized to obtain aerogel-like structures for subsequent characterization.

### 4.5. Frontal Velocity and Temperature Tests

To determine the front velocity, the time required for the polymerization front to advance every 1 cm along the quartz boat was recorded using a stopwatch. The front velocity was subsequently calculated from the slope of the position–time linear fit. Additionally, the temperature profile during the FP process was monitored using an infrared thermal imaging system. Upon initiation and the formation of the front, thermal images were captured at 60 s intervals at a fixed point in the center of the mold, from which the temperature–time curves and *T*_max_ were extracted.

### 4.6. SEM Measurements

The microstructure of the hydrogels was characterized using a scanning electron microscope (SEM, QUANTA 200, FEI, Eindhoven, The Netherlands) operated at an accelerating voltage of 20.0 kV. Prior to imaging, the samples were immersed in deionized water to remove residual solvents and unreacted monomers, with the water refreshed daily until swelling equilibrium was achieved. The fully swollen hydrogels were subsequently freeze-dried for 7 days. The dried samples were sectioned longitudinally to expose their internal structures, sputter-coated with gold and imaged under vacuum to reveal the porous network.

### 4.7. Swelling Ratio Measurements

Gravimetric analysis was employed to monitor the swelling kinetics of the hydrogels. Following the initial weighing (W_0_), each specimen was submerged in deionized water. At specific time points, the samples were temporarily extracted, gently dabbed with filter paper to eliminate residual surface moisture, and weighed again to determine the transient mass (W*_t_*). They were then promptly returned to the water bath. This continuous tracking proceeded until a constant mass was achieved, indicating an equilibrium state. The water uptake capacity was subsequently deduced via the formula below:

(2) $$S\mathrm{welling}\;R\mathrm{atio}(\%)\;=\;\frac{W_{t}-W_{0}}{W_{0}}\times 100\%$$

### 4.8. Tensile Testing

Mechanical evaluation was conducted under ambient conditions employing a SANS CMT6203 universal testing machine (Shenzhen Sans Test. Machine Co. Ltd., Shenzhen, China). Prior to the assay, the hydrogel networks were molded into standard dumbbell-shaped configurations and kept in closed containers to prevent water loss. Uniaxial tension was applied at a steady extension rate of 10 mm/min, yielding stress–strain profiles from which the fracture strain and maximum tensile strength were extracted.

### 4.9. Biocompatibility Evaluation

The biocompatibility of the hydrogels was evaluated using Live/Dead staining and CCK-8 assays. L929 fibroblasts were cultured in complete medium (Dulbecco’s modified Eagle’s medium supplemented with 10% fetal bovine serum) at 37 °C in a humidified atmosphere containing 5% CO_2_. Prior to cell experiments, the hydrogel samples were sterilized by autoclaving and incubated in complete medium at a concentration of 0.1 g/mL for 24 h to obtain extracts. The collected extracts were subsequently diluted with fresh medium for further use.

For Live/Dead staining, L929 cells (3 × 10^4^ cells per well) were seeded in 24-well plates and cultured for 24 h. The medium was then replaced with hydrogel extracts, followed by another 24 h incubation. After washing with PBS, cells were treated with Live/Dead staining solution and incubated in the dark at room temperature for 15 min. Fluorescence images were acquired using an inverted fluorescence microscope (ZEISS Axio Vert.A1, Carl Zeiss, Jena, Germany), where live cells emitted green fluorescence and dead cells showed red fluorescence.

For the CCK-8 assay, cells were seeded and treated under the same conditions as described above. After 24 h incubation, the culture medium was replaced with 100 μL of 10% CCK-8 solution per well and incubated for 2 h at 37 °C in 5% CO_2_. Subsequently, 60 μL of the solution from each well was transferred to a 96-well plate, and the absorbance was measured at 450 nm using a microplate reader (TECAN SPARK 10M, Tecan Austria GmbH, Grödig, Austria).

### 4.10. Antibacterial Activity Evaluation

The antibacterial activity of the hydrogels was evaluated using both optical density (OD) measurements and the standard plate counting method. Prior to testing, the hydrogel samples were sterilized via ultraviolet irradiation for 10 min. A liquid bacterial culture medium was prepared containing peptone, yeast extract and NaCl. Subsequently, 5 μL of *S. aureus* or *E. coli* suspension was inoculated into the medium, followed by the addition of the hydrogel sample. The mixtures were co-cultured at 37 °C for 12 h. A blank control group (bacterial suspension without any hydrogels) was maintained under identical conditions.

At 2 h intervals, a 200 μL aliquot of the suspension was withdrawn to measure the optical density at 600 nm (OD_600_) using a microplate reader. After 12 h of co-incubation, the bacterial suspensions were serially diluted (10^4^-fold for *E. coli* and 10^6^-fold for *S. aureus*) with sterile water. Thereafter, 150 μL of each diluted suspension was spread onto solid agar plates and incubated at 37 °C for an additional 12 h. The resulting bacterial colonies were photographed using a gel imaging system.

### 4.11. Characterization

The chemical structure of the hydrogels was characterized by Fourier transform infrared spectroscopy (FTIR, Thermo Nicolet AVATAR-FTIR-360, Thermo Nicolet Corporation, Madison, WI, USA) over a wavenumber range of 500–4000 cm^−1^ with a resolution of 1 cm^−1^. Prior to analysis, the samples were immersed in deionized water for one week to eliminate residual monomers and solvents, followed by drying at 60 °C to a constant weight. The dried samples were then finely ground with KBr and compressed into pellets for measurement. Thermogravimetric analysis (TGA) was performed using a Netzsch TG209F1 instrument (NETZSCH-Gerätebau GmbH, Selb, Germany) under a nitrogen atmosphere with a flow rate of 250 mL/min. The samples were heated from 30 to 800 °C at a rate of 10 °C/min.

## Data Availability

Data is contained within the article or Supplementary Material. Further inquiries can be directed to the corresponding authors.

## Supplementary Materials

The following supporting information can be downloaded at: https://www.mdpi.com/article/10.3390/gels12060547/s1, Figure S1: (a) Schematic illustration of the GelMA synthesis process; (b) Methacrylation of gelatin for GelMA; (c) ^1^H-NMR spectra of pristine gelatin and GelMA; Figure S2: FTIR spectrum of GelMA; Figure S3: Plot of the front position versus time during the FP process at varying GelMA concentrations; Figure S4: Young’s modulus of the PAAH-GelMA hydrogels with different GelMA mass fractions; Figure S5: Representative agar plate photographs illustrating the antibacterial efficacy of the hydrogels against *E. coli* and *S. aureus*; Figure S6. Infrared thermal images during the attempted FP with mass fractions of F-CDs and GelMA of 1.2% and 5%, respectively; Figure S7: Young’s modulus of hydrogels with different F-CDs mass fractions.
