# Supplementary material for: Frontal Polymerization-Enabled Rapid Fabrication of Gelatin-Containing Hydrogels with Good Mechanical and Biological Properties
Source: Gels. 2026 Jun 19;12(6):547. doi: 10.3390/gels12060547 (PMC13299243; doi:10.3390/gels12060547)
Supplement: Supplementary file 1 [file gels-12-00547-s001.zip › gels-4365643-supplementary.pdf]

gels

Supplementary Materials

Frontal Polymerization-Enabled Rapid Fabrication of Gelatin-Containing Hydrogels with Good Mechanical and Biological Properties

Fucheng Li, Weixiong Yuan, Yonglin Chen, Chang Liu*, Cai-Feng Wang* and Su Chen

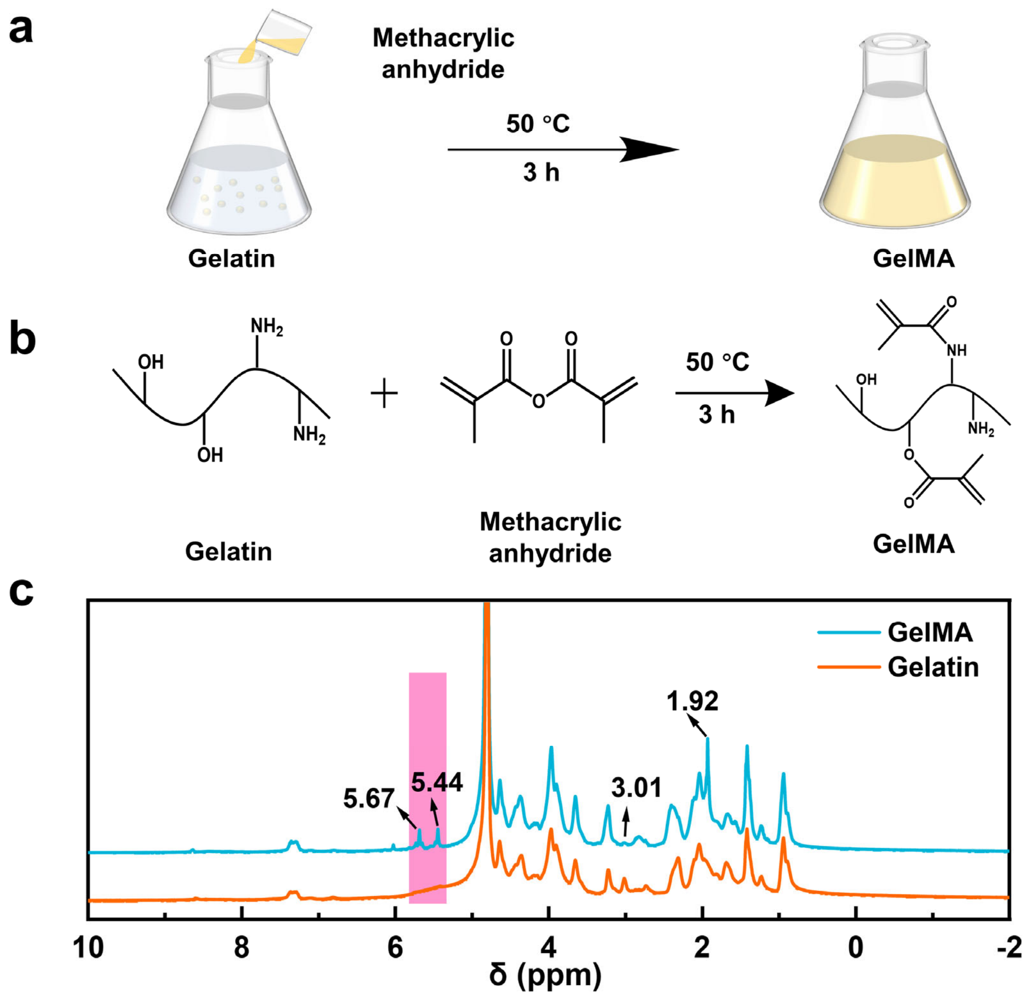

Figure S1. (a) Schematic illustration of the GelMA synthesis process; (b) Methacrylation of gelatin for GelMA; (c) ^{1}H-NMR spectra of pristine gelatin and GelMA.

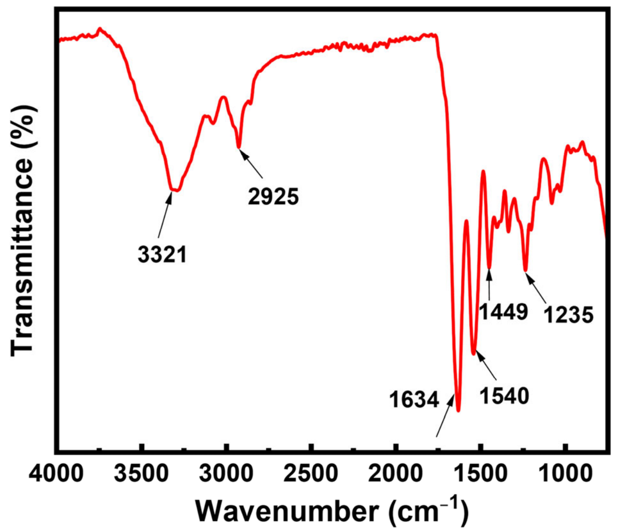

Figure S2. FTIR spectrum of GelMA.

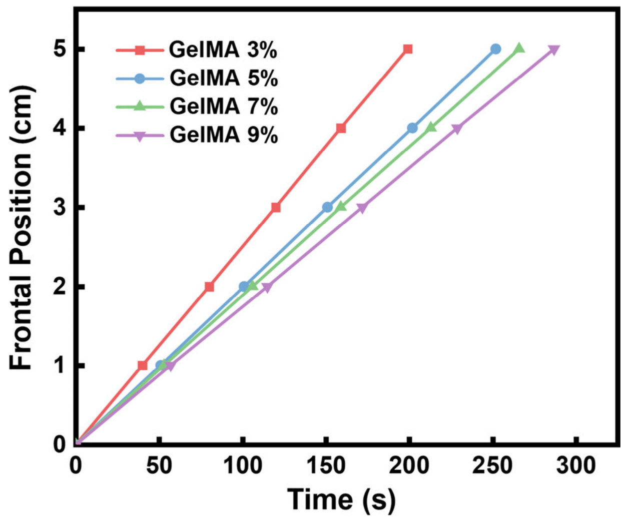

Figure S3. Plot of the front position versus time during the FP process at varying GelMA concentrations.

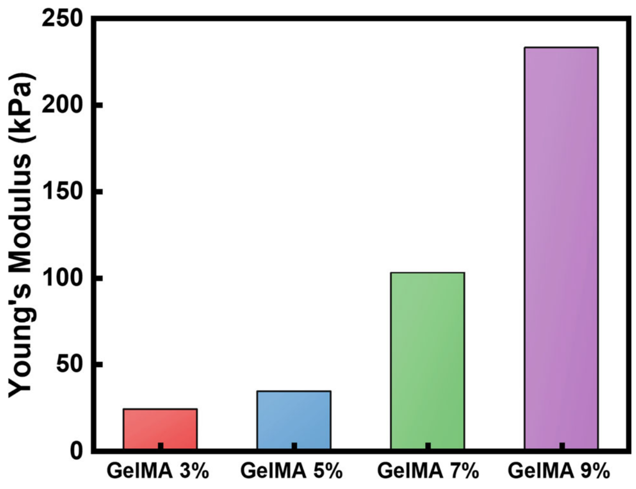

Figure S4. Young's modulus of the PAAH-GelMA hydrogels with different GelMA mass fractions.

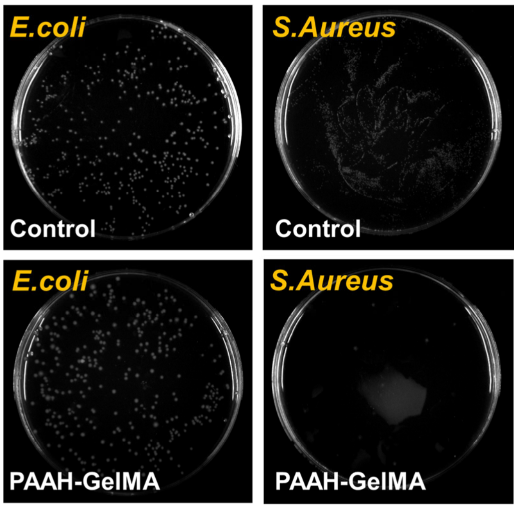

Figure S5. Representative agar plate photographs illustrating the antibacterial efficacy of the hydrogels against *E. coli* and *S. aureus*.

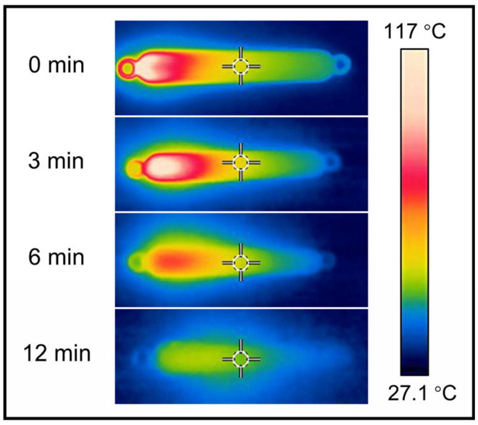

Figure S6. Infrared thermal images during the attempted FP with mass fractions of F-CDs and GelMA of 1.2% and 5%, respectively.

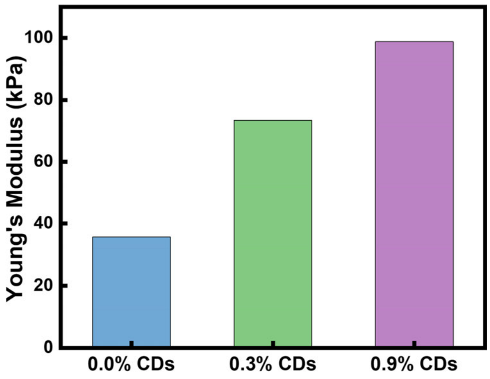

Figure S7. Young's modulus of hydrogels with different F-CDs mass fractions.
